# Clinical Efficacy of Dexamethasone in the Treatment of Patients with Tuberculous Meningitis: A Meta-Analysis

**DOI:** 10.1155/2022/2180374

**Published:** 2022-03-30

**Authors:** Wei Wang, Juan Gao, Jiarui Liu, Jinxi Qi, Qifan Zhang

**Affiliations:** Department of Neurology, People's Hospital of Ningxia Hui Autonomous Region, Yinchuan 750002, China

## Abstract

**Objective:**

This study aimed to systematically evaluate the clinical efficacy of dexamethasone in patients with tuberculous meningitis (TBM) through meta-analysis.

**Method:**

PubMed, Web of Science, Embase, China National Knowledge Infrastructure (CNKI), and the Wanfang Databases were searched, and all relevant Chinese and English literature from 2000 to 2021 were retrieved from each database. We collected randomized controlled trials of conventional antituberculosis drugs combined with dexamethasone treatment (treatment group) and conventional antituberculosis drug treatment or combined with placebo treatment (control group) in TBM patients. Meta-analysis was performed with Stata16.0 software.

**Results:**

A total of 1645 articles were retrieved, and 11 articles were finally included in the study. Meta-analysis results showed that the treatment group had a significantly higher response rate and lower incidence of adverse reactions compared with the control group. Additionally, compared with the control group, the postoperative cerebrospinal fluid cell count, protein content, and glucose in the treatment group were significantly lower, while the chloride level increased.

**Conclusion:**

Conventional antituberculosis drugs combined with dexamethasone therapy can improve cerebrospinal fluid cell count, protein content, glucose, and chloride levels in patients with TBM. This treatment can improve the treatment effective rate and reduce the incidence of adverse reactions, which is considered an effective treatment for TBM. Our results provide strong evidence for enhancing existing treatment regimens and developing novel combination therapy to improve TBM treatment efficacy.

## 1. Introduction

Tuberculous meningitis (TBM) is a nonpurulent inflammation of the meninges caused by the invasion of *Mycobacterium tuberculosis* into the subarachnoid space [1]. The disease can occur alone or secondary to active tuberculosis lesions in other parts of the body, with pulmonary tuberculosis being the most common. This is more common in patients with hematogenous disseminated pulmonary tuberculosis. In rare cases, the disease can also be caused by liquefaction and ulceration rupture of tuberculosis lesions in the brain parenchyma or meninges, allowing a large number of *Mycobacterium tuberculosis* to enter the subarachnoid space [2]. In addition to involving the pia mater, the arachnoid, brain parenchyma, and cerebral blood vessels are also often affected and are the most common and most severe forms of extrapulmonary tuberculosis, accounting for about 70% of neurological tuberculosis [3]. TBM is more common in children, but it also has certain incidences in adults. It is one of the most severe and complex diseases to treat in central nervous system infections. In addition, TBM has a higher mortality rate, with 25% mortality in HIV-negative people and 65% mortality in HIV-positive people [4]. Approximately 50% of survivors may be left with permanent central nervous system damage [5]. Therefore, finding a more effective treatment for TBM is urgent.

At present, there is no recognized and reliable treatment plan for TBM globally. Antituberculosis treatment is the foundation for treating TBM, and the main treatment for TBM is to learn from that for pulmonary tuberculosis. The WHO-recommended treatment regimen for pulmonary tuberculosis is 2HRZE/4HR, and this regimen is also applicable in TBM [6]. However, this short-term regimen of 6 months has been reported to be associated with higher relapse rates and higher incidences of neurological sequelae. The British Infection Society recommends that the course of treatment for TBM should be at least 1 year, and most Chinese scholars also support it [7]. A consensus has been reached on the use of hormones in combination with conventional treatment in TBM. A large-scale meta-analysis suggested that the use of hormones could reduce the mortality of patients with TBM and the incidence of neurological sequelae in surviving patients [8]. Additionally, Thwaites et al. observed the role of hormones in TBM patients by MRI and found a reduction in the occurrence of hydrocephalus and infarction after receiving such drugs [9].

TBM treatment should include supplementary corticosteroids, according to international standards [7]. Although the best corticosteroid formulation, dosage, and route of administration are uncertain, corticosteroid usage in TBM is frequent [8, 9]. Dexamethasone is routinely used since it is inexpensive and easily accessible. Issues with extended intravenous therapy, availability to intravenous therapy, and pill burden for oral medication all demand attention when novel corticosteroid therapies are introduced in clinical practice [8]. These drugs are expected to help reduce inflammation of the surface of the brain and related blood vessels, as well as lower pressure inside the brain, and thus reduce the risk of death [9]. As a glucocorticoid, dexamethasone has anti-inflammatory effects and can relieve brain edema. Many scholars believe that the combination of dexamethasone and conventional antituberculosis treatment can maximize the life and health of patients with TBM [8]. However, there are few reports on this combination in TBM patients. Besides, the sample size of a single study is small, also with inconsistent results among individual studies. Given the lack of systematic evaluation on the effectiveness of dexamethasone combined with conventional treatment for TBM, some doctors are afraid that glucocorticoids may increase survival but result in more severely disabled survivors. As a result, this type of combination treatment is rarely employed in clinical practice. This study systematically analyzed the existing randomized controlled trials (RCTs) using meta-analysis. We aim to provide an evidence-based medicine basis for the combined therapy for TBM, as well as innovation for improving current treatment options.

## 2. Materials and Methods

### 2.1. Literature Search

Literature searches were conducted through PubMed, Web of Science, Embase, China National Knowledge Infrastructure (CNKI), and Wanfang Database. Articles published between 2000 and 2021 were retrieved, with the search term set as follows: (#1 “Dexamethasone”) and (#2 “Tuberculous”) and (#3 “Meningitis” or “Tuberculous Meningitis” or “TBM”).

### 2.2. Screening Criteria

Inclusion criteria were as follows: (1) study design: RCTs, controlled clinical trials, and cohort studies; (2) study subjects: all patients who met the clinical TBM diagnostic criteria [10] and have relevant symptoms; patients with a history of other pulmonary tuberculosis diseases; (3) intervention: the treatment group was treated with conventional antituberculosis drugs combined with dexamethasone; the control group was treated with conventional antituberculosis drugs or combined with placebo treatment; (4) outcome measures: treatment response rate, incidence of adverse reactions, cerebrospinal fluid cell count after treatment, cerebrospinal fluid protein content after treatment, cerebrospinal fluid glucose level after treatment, chloride level after treatment, and other indicators; (5) informed consent: the family members of the patients were informed about the study and signed the informed consent.

Exclusion criteria were as follows: (1) reviews, conferences, review articles, case reports, or abstracts; (2) duplicate publications; (3) inability to obtain information and data from original articles.

### 2.3. Data Extraction

Two reviewers independently screened the literature according to the inclusion and exclusion criteria and then extracted and sorted the relevant data. For literature screening, the titles, content, research methods, and abstracts of retrieved articles were reviewed, and those who met the inclusion criteria were selected. Disagreements were resolved through discussion or consultation with a third reviewer. The data extracted mainly included (1) basic information in the literature, (2) type of research method, (3) sample size, and (4) outcome indicators.

### 2.4. Statistical Analysis

Meta-analysis was performed using Stata16. Standardized mean difference (SMD) was used as effect size for measurement data, and the odds ratio (OR) and 95% confidence interval (CI) were used for categorical variables. The I^2^ statistic was used to quantify heterogeneity; the random-effects model was used to combine the effect sizes if I^2^>50% and *P* < 0.05, which suggested significant heterogeneity across studies; otherwise, the fixed-effects model was used. Sensitivity analysis tested the stability of the overall results of the meta-analysis. *P* < 0.05 indicated that the difference was statistically significant.

## 3. Results

### 3.1. Literature Screening Results

**Figure 1:**
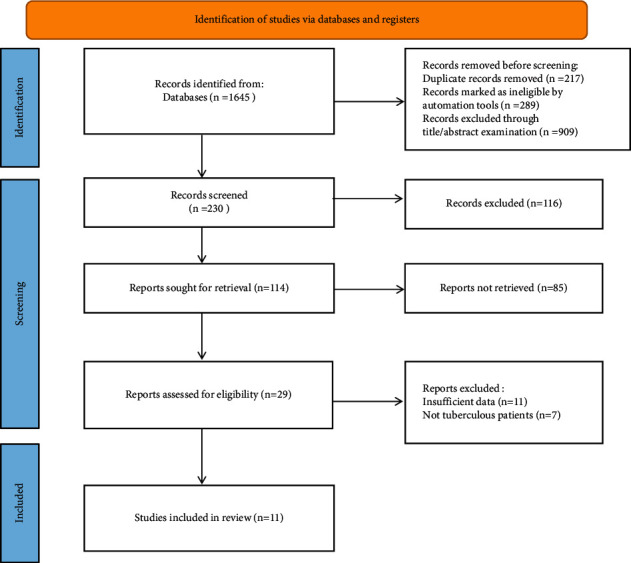
Flowchart of literature screening.

A total of 1645 articles were retrieved, 217 duplicate articles were excluded, and 289 articles marked as unqualified by automated tools were removed. Then, 909 articles were excluded by examining titles/abstracts. The remaining 230 articles' full text was read through, and 219 articles that did not meet the inclusion criteria were excluded. Eleven studies were finally included [1, 11–20]. The literature screening process is shown in Figure 1. The characteristics of the included studies are shown in Table 1.

### 3.2. Comparison of Effective Rate

**Figure 2:**
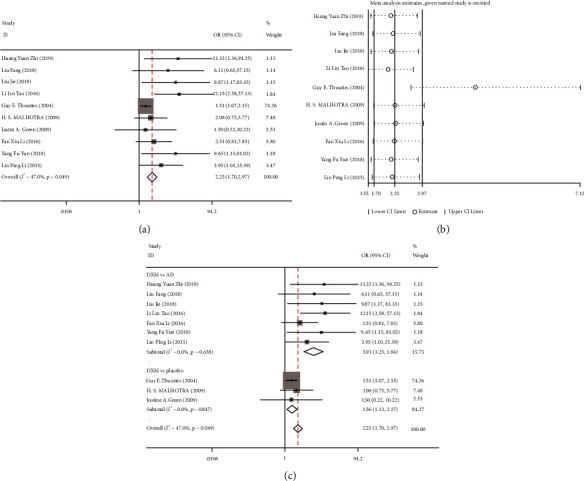
Meta-analysis of the treatment response rate of dexamethasone in the treatment of tuberculous meningitis (TBM) patients. (a) Forest plot of the effect of dexamethasone combined with antituberculosis drugs on the treatment response rate of TBM patients; (b) sensitivity analysis of the treatment response rate; (c) subgroup analysis of the effect of dexamethasone on the treatment response rate of TBM patients.

Totally, 10 [1, 11–19] studies reported the effective rate, and the fixed-effects model was used to combine the effect size (I^2^ = 47.0%, *P* = 0.049). The results showed that the treatment effective rate of the treatment group was significantly higher than that of the control group (OR = 2.25, 95% CI: 1.71–2.97, *P* < 0.001) (Figure 2(a)). Furthermore, the sensitivity analysis by the one-by-one elimination method showed that the study by Guy E. Thwaites (2004) [21] possibly affected the heterogeneity, but the statistical results of the effect size of this study were stable after its exclusion (Figure 2(b)). Next, this study conducted a subgroup analysis on the effective rates of different treatment methods; 7 studies compared dexamethasone with antituberculosis drugs, and 3 studies compared dexamethasone with placebo treatment. The results showed that the effective rate of the dexamethasone group was better than that of the conventional antituberculosis drug group (OR = 5.93, 95% CI: 3.25–10.84, *P* < 0.001) and the placebo group (OR = 1.56, 95% CI: 1.13–2.2, *P* = 0.007) (Figure 2(c)).

### 3.3. Comparison of the Incidence of Adverse Reactions

**Figure 3:**
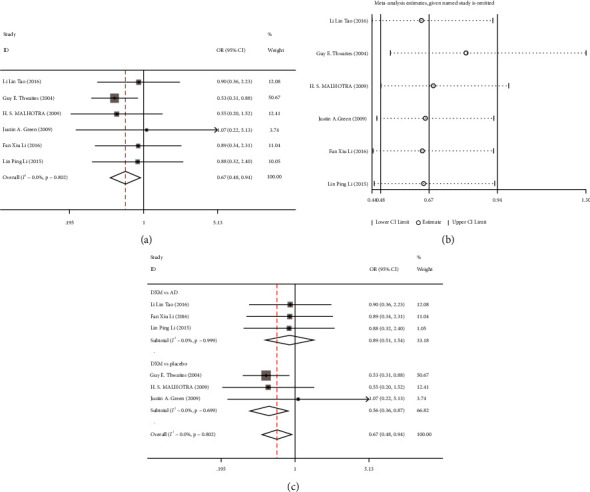
Meta-analysis of the incidence of adverse reactions of dexamethasone in patients with tuberculous meningitis (TBM). (a) Forest plot comparing the incidence of adverse reactions of dexamethasone combined with antituberculosis drugs in patients with TBM; (b) sensitivity analysis of the incidence of adverse reactions; (c) subgroup analysis of the incidence of adverse reactions of dexamethasone in patients with TBM.

Six [1, 11–15] studies reported the effect of dexamethasone on the incidence of adverse reactions in patients with TBM. Since there was no significant heterogeneity in the included studies (I^2^ = 0.00%, *P* = 0.802), the fixed-effects model was used to combine the effect size. The results demonstrated that the incidence of adverse reactions in the treatment group was significantly lower than that in the control group (OR = 0.67, 95% CI: 0.48–0.94, *P* = 0.022) (Figure 3(a)). Sensitivity analysis showed that the study by Guy E. Thwaites (2004) [1] might affect the heterogeneity, but the statistical results of the effect size were stable after its exclusion (Figure 3(b)). Subsequently, we performed a subgroup analysis of the adverse reaction rates of different drug groups; 3 studies compared dexamethasone with antituberculosis drugs, and 3 studies compared dexamethasone with placebo treatment. Subgroup analysis results showed that the incidence of adverse reactions in the dexamethasone group was better than that in the conventional antituberculosis drug group (OR = 0.89, 95% CI: 0.51–1.54, *P* = 0.67) and the placebo group (OR = 0.56, 95% CI: 0.36–0.87, *P* = 0.01) (Figure 3(c)).

### 3.4. Meta-Analysis of Biochemical Index Levels in Cerebrospinal Fluid of TBM Patients

**Figure 4:**
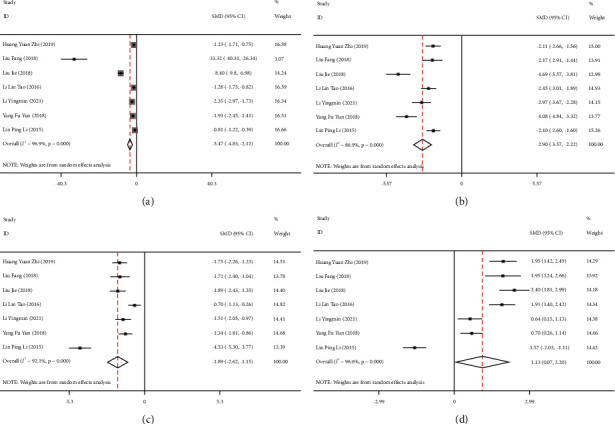
Forest plots comparing the levels of biochemical parameters in cerebrospinal fluid of patients with tuberculous meningitis (TBM) treated with dexamethasone. (a) Cell count; (b) protein level; (c) glucose level; (d) chloride level.

Seven [11, 15–20] studies reported the effects of dexamethasone on cell count, protein, glucose, and chloride levels in cerebrospinal fluid of TBM patients. The included studies were tested for heterogeneity (cell count: I^2^ = 96.9%, *P* < 0.001; protein: I^2^ = 86.9%, *P* < 0.001; glucose: I^2^ = 92.1%, *P* < 0.001; chloride: I^2^ = 96.6%, *P* < 0.001), and the random-effects model was used to combine the effect size due to the significant heterogeneity. The results showed that (Figures 4(a)–4(d)), after treatment, the cerebrospinal fluid cell count (SMD = −3.46, 95%CI: −4.83 to −2.12, *P* *<* 0.001), protein content (SMD = −2.90, 95% CI: −3.57 to −2.22, *P* < 0.001), and glucose (SMD = −1.89, 95% CI: −2.62 to −1.15, *P* < 0.001) in the treatment group were significantly lower than those in the control group, while the chloride levels were significantly increased (SMD = 1.13, 95% CI: 0.07–2.20, *P* = 0.04).

**Figure 5:**
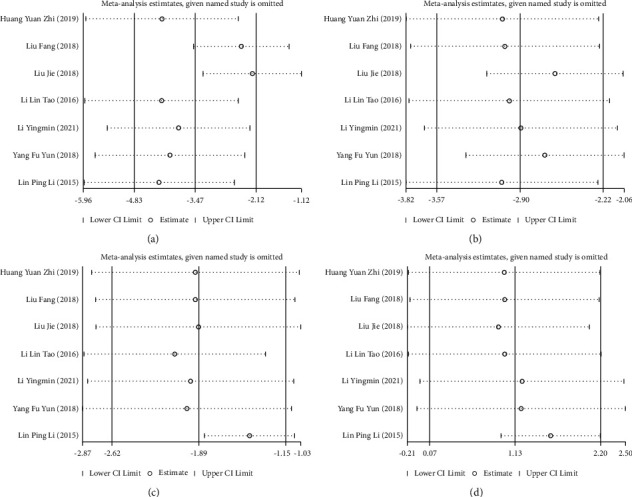
Sensitivity analysis of the effect of dexamethasone on the levels of biochemical parameters in cerebrospinal fluid of patients with tuberculous meningitis (TBM). (a) Cell count; (b) protein level; (c) glucose level; (d) chloride level.

Further sensitivity analysis was required and conducted through the one-by-one elimination method; the analysis results found that the study by Lin Pingli (2015) [15] and the study by Liu Jie (2018) [17] were the primary sources of increased heterogeneity. After excluding these 2 articles, the obtained results were similar to previous results. Therefore, the results of this study were relatively stable and reliable (Figures 5(a)–5(d)).

## 4. Discussion

As the most common type of neurological tuberculosis, TBM is an intracranial lesion formed by *Mycobacterium tuberculosis* invading the subarachnoid space, subsequently infecting the pia mater and arachnoid, and further involving the brain parenchyma and cerebral blood vessels. TBM is one of the most severe forms of tuberculosis with high mortality, long disease duration, and severe sequelae [21]. Also, this disease has higher morbidity and mortality in HIV-infected or drug-resistant patients. Its common clinical manifestations are malaise, fatigue, anorexia, vomiting, headache, and fever [22, 23]. The presence of TBM affects the limb function or neurological function of patients and was even life-threatening in severe cases. In short, TBM will reduce the quality of life of patients.

Currently, TBM is mainly treated with antituberculosis drugs, mainly isoniazid, pyrazinamide, and rifampicin. Such drugs can effectively inhibit the activity of *tuberculosis* bacteria, thereby limiting their spread and achieving the goal of elimination [17]. However, they have certain limitations in controlling inflammation, and during the application process, patients may experience relatively more adverse reactions that seriously reduce the patient's medical experience. Dexamethasone is a synthetic adrenal cortical hormone that can reduce vascular permeability, relieve cerebral edema and hypertension symptoms, accelerate cerebral blood supply, and promote brain metabolism. At the same time, dexamethasone can inhibit the synthesis of inflammatory mediators to reduce their activity, thereby decreasing inflammation [24]. In this regard, dexamethasone as adjuvant therapy is proposed in clinical practice. Studies have shown that dexamethasone can effectively reduce toxic symptoms in patients, relieve cerebral edema, and prevent subarachnoid obstruction in some cases [25]. Based on the above findings, our study further clarified the clinical efficacy of dexamethasone combined with conventional treatment of TBM. We conducted a meta-analysis and compared the difference in the clinical efficacy between dexamethasone combined with conventional treatment and conventional treatment alone or combined with placebo treatment in TBM patients. In this study, a total of 11 studies were included for meta-analysis, and the results showed that dexamethasone combined with the conventional antituberculosis drug treatment could achieve better results in the treatment response rate and the incidence of adverse reactions.

Cerebrospinal fluid analysis is a key link in the diagnosis of TBM, and typical findings include increased lymphocytosis and protein and decreased glucose [26, 27]. Previous clinical studies have clearly demonstrated three aspects of successful TBM treatment [28]: (1) effective antimicrobial therapy, (2) control of host inflammatory response, and (3) supportive intervention to reduce elevated intracranial pressure. This means that effective antituberculosis treatment in the acute phase of TBM and improvement of critical condition as soon as possible are the keys to improving the survival rate of TBM patients and reducing sequelae. In this study, we found that the cerebrospinal fluid cell count, protein content, and glucose levels were significantly decreased in the two groups of patients after treatment while the chloride levels were increased. However, the treatment group had a better outcome than the control group, indicating the clinical effect of dexamethasone combined with antituberculosis drugs is relatively good.

There are still some limitations in this study: (1) this study only included 11 articles for meta-analysis, and the sample size was small; (2) no subgroup analysis was carried out on drug selection, dosage, frequency, and course of treatment in the included studies, so there is inevitably some heterogeneity; (3) only 3 of the included studies were foreign studies, and the rest were all domestic studies. The quality of literature was not very high in the comprehensive evaluation, and the statistical results may be biased. Therefore, large-sample, high-quality, multicenter RCTs are required to enhance the accuracy and credibility of the research results and to provide more effective clinical treatments.

## 5. Conclusion

In conclusion, dexamethasone combined with conventional antituberculosis drug therapy can improve the effectiveness of TBM treatment and reduce the incidence of adverse reactions. Such combined treatment can effectively adjust the cerebrospinal fluid cell count, protein content, glucose, and chloride levels and has improved efficacy.

## Figures and Tables

**Table 1:** Basic characteristics of the included studies.

Study	Year	Sample time (year.month)	Cases (Treat/Con)	Age (years)		Sex (male/female)		Measures (Con group)	Study design	Outcome measures
				Treat group	Treat group	Treat group	Con group			
Huang Yuanzhi	2019	2018.01–2019.01	40/40	48.5 ± 3.3	48.5 ± 3.3	21/19	22/18	Isoniazid, rifampin, ethambutol, pyrazinamide	RCT	①③④⑤⑥
Liu Fang	2018	2014.01–2016.01	23/23	57.7 ± 2.4	57.9 ± 2.4	12/11	13/10	Isoniazid, rifampin, ethambutol, pyrazinamide	Retrospective	①③④⑤⑥
Liu Jie	2018	2016.08–2017.08	38/38	20–78	21–78	21/17	22/16	Isoniazid, rifampin, ethambutol, pyrazinamide	RCT	①③④⑤⑥
Li Lintao	2016	2015.05–2016.04	43/43	43.4 ± 1.8	42.1 ± 1.6	23/20	21/22	Isoniazid, rifampin, ethambutol, pyrazinamide, mannitol	RCT	①②③④⑤⑥
Li Yingmin	2021	2018.02–2020.06	34/34	40.8 ± 4.3	40.1 ± 4.2	19/15	18/16	Isoniazid, rifampin, ethambutol, pyrazinamide	RCT	③④⑤⑥
Fan Xiu Li	2016	2014.07–2015.10	53/53	48.2 ± 3.3	48.0 ± 3.7	30/23	29/24	Isoniazid, rifampin, ethambutol, pyrazinamide, mannitol	RCT	①②
Yang Fuyun	2018	NP	42/42	33.9 ± 4.3	34.8 ± 4.1	22/20	25/17	Isoniazid, rifampin, levofloxacin	NP	①③④⑤⑥
Lin Pingli	2015	2014.02–2015.01	48/48	40.1 ± 4.6	40.4 ± 4.1	20/28	21/27	Isoniazid, rifampin, ethambutol, pyrazinamide, mannitol	RCT	①②③④⑤⑥
Guy E. Thwaites	2004	2001.04–2003.03	274/271	15–88	15–84	168/106	163/108	Isoniazid, rifampin, pyrazinamide, placebo	RCT	①②
H. S. Malhotra	2009	2006.01–2007.07	31/30	15–66	15–70	15/16	14/16	Isoniazid, rifampin, ethambutol, pyrazinamide, placebo	Retrospective	①②
Justin A. Green	2009	2001.04–2003.03	18/19	19.3–40.5	23.0–44.0	8/10	7/12	Isoniazid, rifampin, pyrazinamide, streptomycin, placebo	Retrospective	①②

Treat, treatment; Con, control; RCT, randomized controlled trial; NR, not reported; ①, effective rate; ②, adverse effects rate; ③, comparison of cerebrospinal fluid cell count after treatment; ④, comparison of protein content in cerebrospinal fluid after treatment; ⑤, comparison of glucose levels in cerebrospinal fluid after treatment; ⑥, comparison of chloride levels after treatment.

## Data Availability

The data used to support the findings of this study are available from the corresponding author upon request.
